# Integrated Rehabilitation of Longitudinal Extensive Transverse Myelitis: Study of a Complex Case

**DOI:** 10.7759/cureus.68778

**Published:** 2024-09-06

**Authors:** Muskan G Qureshi, Pallavi Harjpal, Anjali V Nawkhare, Akshaya Saklecha, Nikita H Seth

**Affiliations:** 1 Department of Neuro Physiotherapy, Ravi Nair Physiotherapy College, Datta Meghe Institute of Higher Education and Research, Wardha, IND

**Keywords:** bilateral upper-lower limb weakness, longitudinal extensive transverse myelitis, magnetic resonating imaging, muscle tone, physiotherapy intervention

## Abstract

Longitudinal extensive transverse myelitis (LETM) is a rare neurological disorder portrayed by inflammation of the spinal cord spanning three or more vertebral segments. It can lead to severe symptoms such as weakness, sensory abnormalities, and dysfunction in various parts of the body. LETM can be idiopathic or associated with autoimmune conditions like Multiple Sclerosis (MS) and Neuromyelitis Optica (NMO). Diagnosis of LETM requires MRI imaging of the spine, and treatment often involves corticosteroids, intravenous immunoglobulins (IVIG), and plasma exchange. Physical therapy plays a crucial role in managing LETM, focusing on improving functional abilities, mobility, and quality of life. This study outlines a 62-year-old male who was admitted with a complaint of bilateral weakness of both upper and lower limbs, predominantly on the left side, seizures, falls, and stiffness of the left limbs. He also complained of a cough with sputum, cluster headache, bowels and bladder dysfunction, and impaired vision. Neurologic examination showed hypotonia and reduced muscle strength in all four limbs with impairment of the optic nerve. The following investigations were conducted: MRI, Chest X-ray, and ultrasound. He was advised for physiotherapy, after which he showed improvement in functional independence and a general recovery following the treatment.

## Introduction

Longitudinal extensive transverse myelitis (LETM) is a rare and enigmatic neurological disorder that involves spinal cord lesions evident on a spine Magnetic resonance imaging (MRI) that encompasses three or more vertebrae [1]. Usually, it affects the thoracic and cervical segments. Apart from this subset, known as idiopathic, it can also be the initial indication of autoimmune conditions like Multiple Sclerosis (MS) and Neuromyelitis Optica (NMO). Even though LETM is traditionally linked to Neuromyelitis Optica Spectrum Disorder (NMOSD), it may additionally have other causes, such as acute disseminated encephalomyelitis (ADEM), systemic lupus erythematosus, antiphospholipid antibody syndrome (APAS), myelin oligodendrocyte glycoprotein antibody disease (MOGAD) determining the origin of the illness which is crucial for long-term care of patient [2]. It was primarily studied in populations of Westerners [3]. A poor prognosis is expected due to the unexpected beginning of variable-aetiology motor, sensory, and autonomic dysfunction [4]. The proportion of sexes turned out 1:2.8 (male: female) in the case of longitudinally extensive transverse myelitis [5]. The clinical features of LETM are as follows: Individuals may exhibit sensory abnormalities and dysfunction (which may include dysregulation of body temperature, lower back pain, urinary incontinence, dysphagia, headache, impairment of vision (due to involvement of the optic nerve), hypotonia, bilateral paraesthesia over the hands and legs with bladder and bowel dysfunction [6].

The pathophysiological basis for LETM often includes demyelination, axonal injury, and immune cell activation within the central nervous system, which is significantly influenced by the aquaporin-4 antibody. Patients with recurrent LETM who have been diagnosed with positive anti-aquaporin-4 antibody counts are more likely to convert to NMO; as a result, they are now referred to as having NMOSD [7]. All cases of LETM should have their MRI of the brain evaluated by the radiologist once compressed myelopathy Is ruled out, as this can help narrow the differential diagnosis [8]. Acute demyelinating diseases have demonstrated a benefit from plasma exchange (PE), which is being used more often in conditions involving humoral factors. It is also helpful in the condition of spinal attacks in extensive transverse myelitis (ETM) [9]. Intra Venous Immune Globulins (IVIG) are frequently used to treat immunodeficiencies and autoimmune disorders [10]. Methylprednisolone administered intravenously is the standard of care [11]. In addition to multiple sclerosis, Guillain-Barré syndrome (GBS), NMOSD, disc hernia, spinal canal stenosis, spinal epidural/subdural bleeding, and spinal tumours are among the differential diagnoses for longitudinal extended transverse myelitis [12] [13]. Physical therapy plays a crucial role in LETM management, aiming to improve functional abilities such as walking, bed mobility, stair climbing, balance, and muscle strength. Patients may also receive cryotherapy, passive range of motion exercises, passive stretching, and muscle stimulation techniques [12]. A comprehensive home exercise program is also recommended to reinforce strength and functionality [14]. The aim of this case report is to present a detailed clinical and diagnostic journey of a patient with LETM, highlighting the complexity of its diagnosis, the therapeutic interventions employed, and the patient's response to treatment.

## Case presentation

A 62-year-old male patient came to the neurology ward with complaints of weakness in the bilateral upper limb and lower limb. The patient reported a history of falls on the left side during an episode of seizure, causing pain and stiffness over the left upper and lower extremities. The patient has been experiencing seizure and quadriparesis for a month, where the left is more affected than the right extremity. The patient also reported a cough with sputum, cluster headache, bowel bladder dysfunction and impaired vision. He was admitted to the neurology ward, where the following investigations were done: magnetic resonance imaging (cervical spine, whole spine, brain screening), chest X-ray, ultrasound of abdominal and pelvic, complete blood count, and routine examination. Physiotherapy was recommended for the patient to improve functional independence and gain functional recovery.

Prior to beginning the examination, the patient's relative gave their consent. The patient had a mesomorphic build, and his vitals were normal. He had hypotonia in both his upper and lower limbs. The patient's mini-mental state examination score (MMSC) was 26, which is normal. The patient was placed supine, lying with his head end raised at a 30° angle for the examination. It was noted that a Foley catheter was present. On sensory examination, the optic nerve (cranial nerve-II) was found to be impaired. As opposed to this, a motor examination indicated a slight decrease in muscle tone in both upper and lower limbs, which was accompanied by little resistance for the remaining (less than half) of the range of motion (based on the tone grading scale) indicated in Table 1. The strength of the muscles of the left extremity and the right extremity were reduced. The patient was functionally incapable of sitting and standing on her own without assistance.

**Table 1:** Muscle tone according to the tone grading scale

Extremities	Pre-intervention Right Limb	Pre-intervention Right Limb
Upper Limb	1+	1+
Lower Limb	1+	1+

Reflexes before the intervention were as follows: On the right side, the biceps, triceps, supinator, knee, and ankle reflexes were normal; plantar reflex was absent. Left side: the biceps, triceps, and supinator reflexes were present and normal; knee and ankle reflexes were absent, as well as the plantar reflex indicated in Table 2. 

**Table 2:** Table demonstrates the variation in reflexes grading

Reflexes Preintervention	Bicep Reflexes	Triceps Reflexes	Supinator Reflexes	Knee Reflexes	Ankle Reflexes	Plantar Reflexes
Right	+	+	+	+	+	Ab
Left	+	+	+	+	Ab	Ab

Investigatory finding

**Figure 1:**
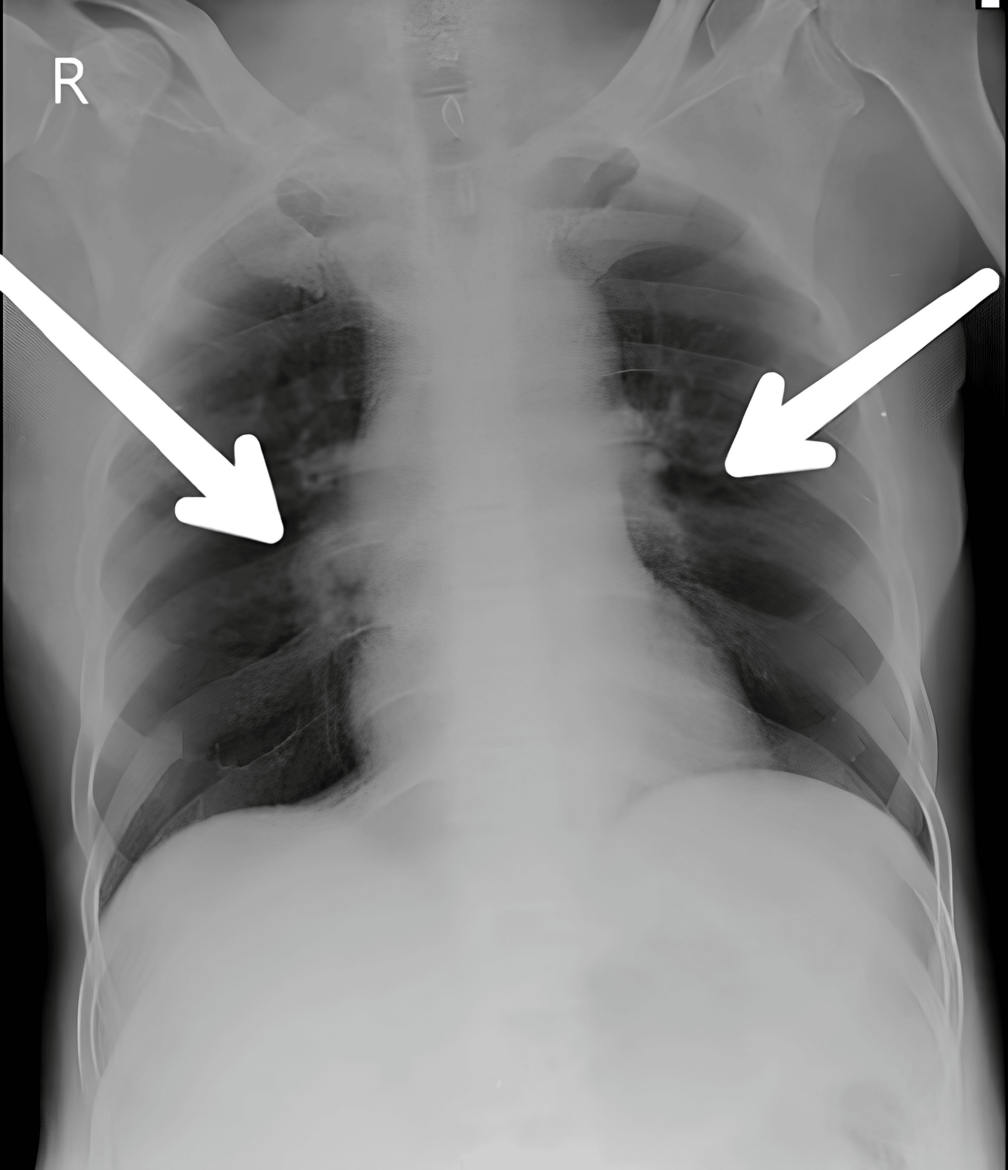
X-ray showing bronchovasicular marking (shown with white arrow)

X-ray findings included bronchovesicular markings, which are nonspecific findings from an overall increase in the density of the lung field or consolidation. Bronchovesicular markings encompass markings due to the visibility of the bronchi and surrounding lung structures in the radiogram; these may become exaggerated in a host of pathologic states. These may be indicative that the normal air-filled lung tissues have been replaced or compressed by inflammation, infection, or fluid collection within the lungs. It is indicated in Figure 1.

**Figure 2:**
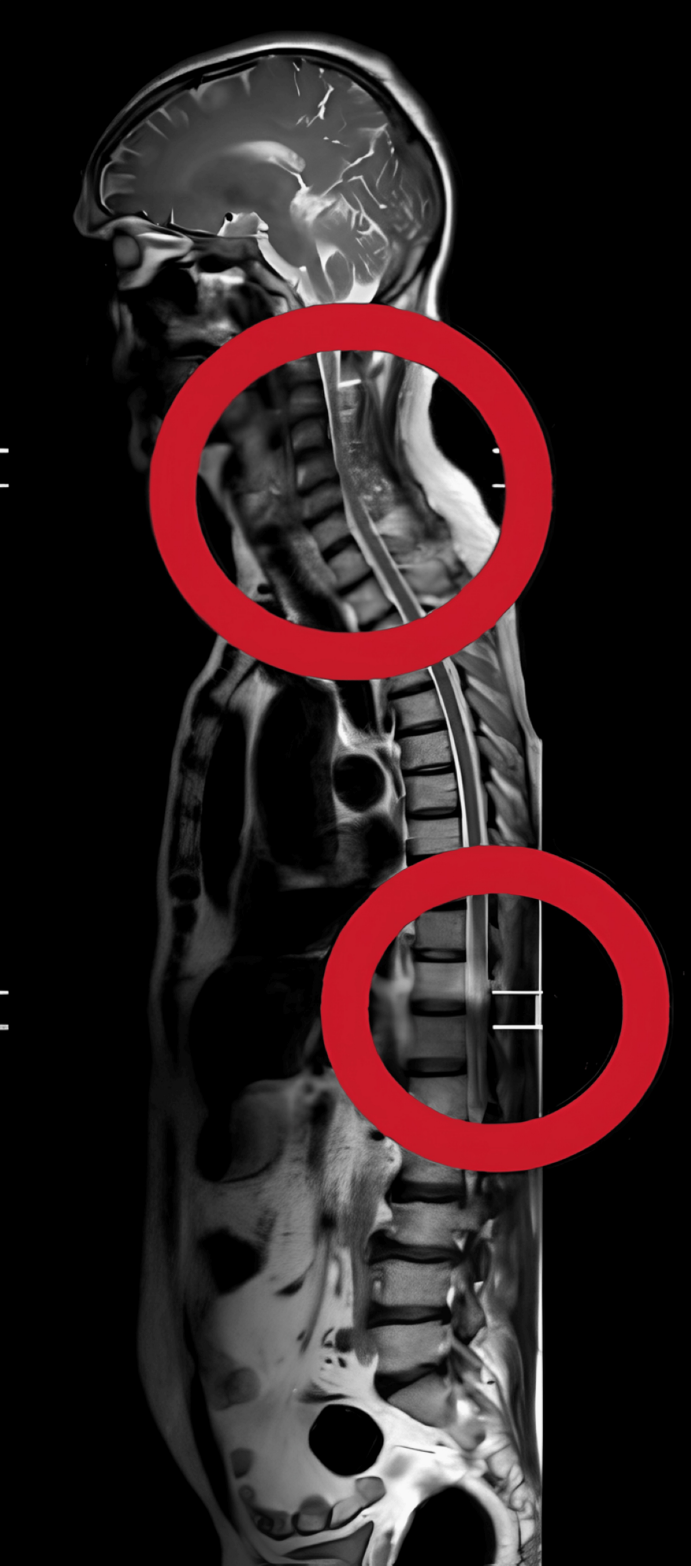
MRI showing compression at the cervical and lumbar region (shown with red circles)

The MRI revealed compression areas in both the cervical and lumbar regions of the spinal cord. This would tend to indicate that the inflammation or swelling affects more than one segment of the spinal cord. Generally, LETM is differentiated by the fact that a long segment of the spinal cord is involved. The MRI findings most likely reveal one continuous area of abnormal signal extending over several vertebral segments, reflective of widespread inflammation, as shown in Figure 2. 

Therapeutic intervention

Early rehabilitation started as soon as the patient was admitted to the hospital. The rehabilitation aims to increase the patient's muscle strength and functional independence, which would enhance their quality of life. The main course of treatment involved intravenous immunoglobulins such as lignocaine, gabapentin, ascorbic syrup, and meropenem. Basic management was provided for weakness through strengthening. A brief overview of the patient's physiotherapy intervention is provided in Table 3.

**Table 3:** Physiotherapy Intervention PROM: Passive range of motion, PNF: Proprioceptive neuromuscular Facilitation, Reps: Repetition, Sec: Second, UL.: Upper Limb, LL.: Lower limb, ACBT.: Active cycle breathing techniques, AAROM: Active assisted range of motion

Problem list	Goals	Interventions	Dosage
Pain	To decrease pain	Hydrocollator pack at the cervical and lumbar region	10-15 min 2-3 times daily
Difficulty in doing transition	To make the patient independent in changing position and posture	Bed mobility exercise From supine-side lying – to bedside sitting Progression – sitting on a stool without support	According to the patient's tolerance
Difficulty in moving upper & lower Limb	To increase the range of motion	AAROM for UL. & LL. Progression active exercise and active range of motion	10 reps in one set
Reduce strength	To increase muscle strength of bilateral upper and lower Limb	Exercises for strengthening: -Incremental resistance band flexion on D1, D2 : -Flexion-Extension pattern of PNF (rhythmic initiation) and dynamic quadriceps. Active range of motion with the resistance of (weight cuff of 500 gm)	10 reps in one set For 10-15 min
Decrease muscle tone	To increase muscle tone	Fast tapping and joint approximation are given from Rood's approach	10 reps in 1 set
Urinary incontinence	To increase pelvic floor muscle strength	Kegels exercises	10 reps in 1 set
Risk of fall	To maintain balance	Instruction in balance maintenance and unsupported bedside sitting. Long periods of sitting and using a wheelchair are additional practices for maintaining balance while standing. A wobble board is used.	10 reps in one set
Difficulty in walking	Gait training	Via Parallel bar Walk on a treadmill and walk through obstacles to advance in your gait training.	10 min initially Increases in time according to patient’s tolerance
Difficulty in breathing & cough	To improve pulmonary function	Deep breathing, ACBT & Incentive spirometer, Breathing exercises with arm movements	10 reps in 1 set

**Figure 3:**
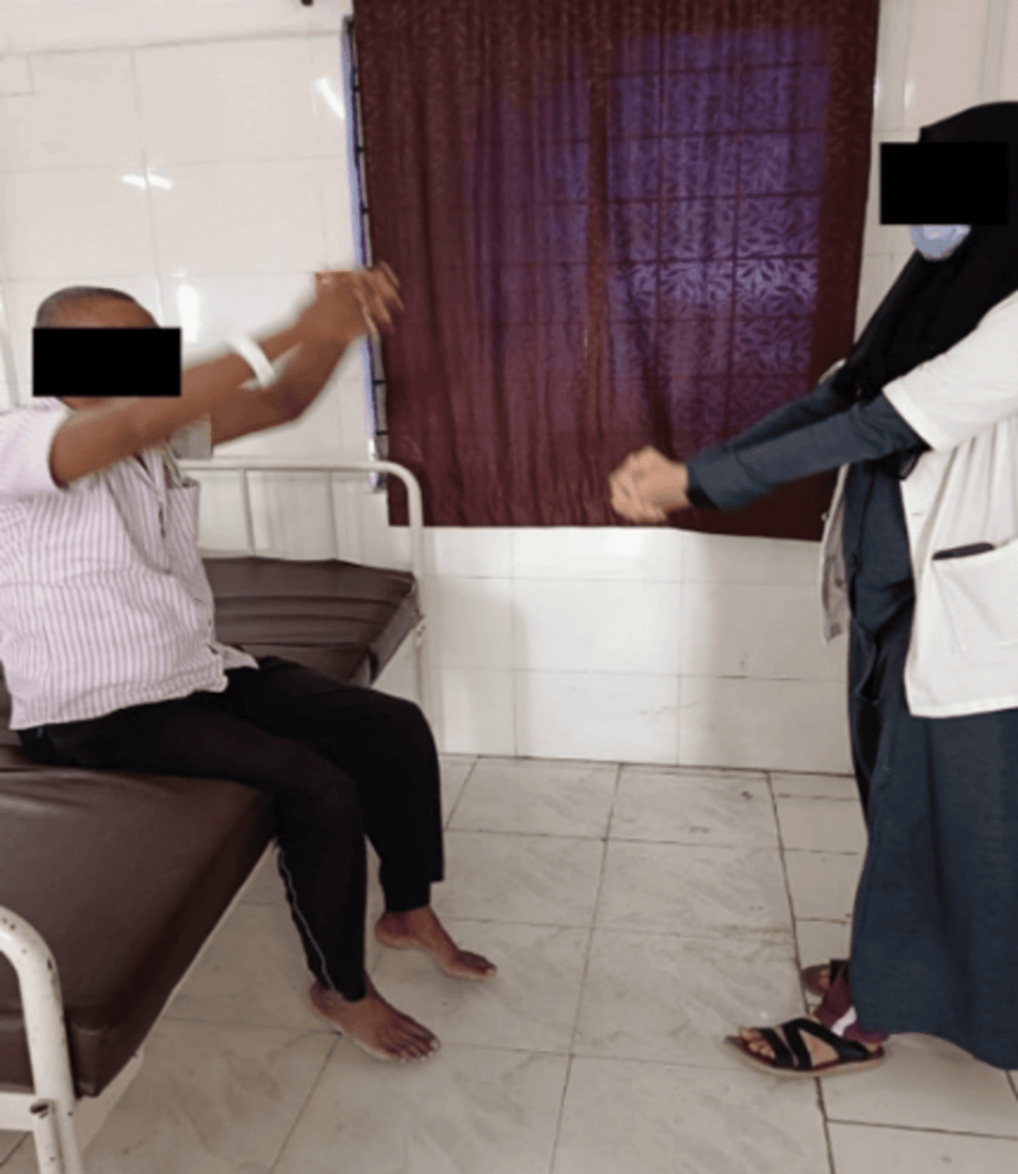
Patient performing breathing exercises with arm movements

Breathing exercises with arm movements were initiated. Here, the patient sits erect at the edge of your bed or in a firm chair and reach arms overhead to create a big stretching yawn. This exercise helps increase coordination, builds strength in the arms and shoulders, and opens up the muscles in your chest so the diaphragm has space to expand. It is shown in Figure 3. 

**Figure 4:**
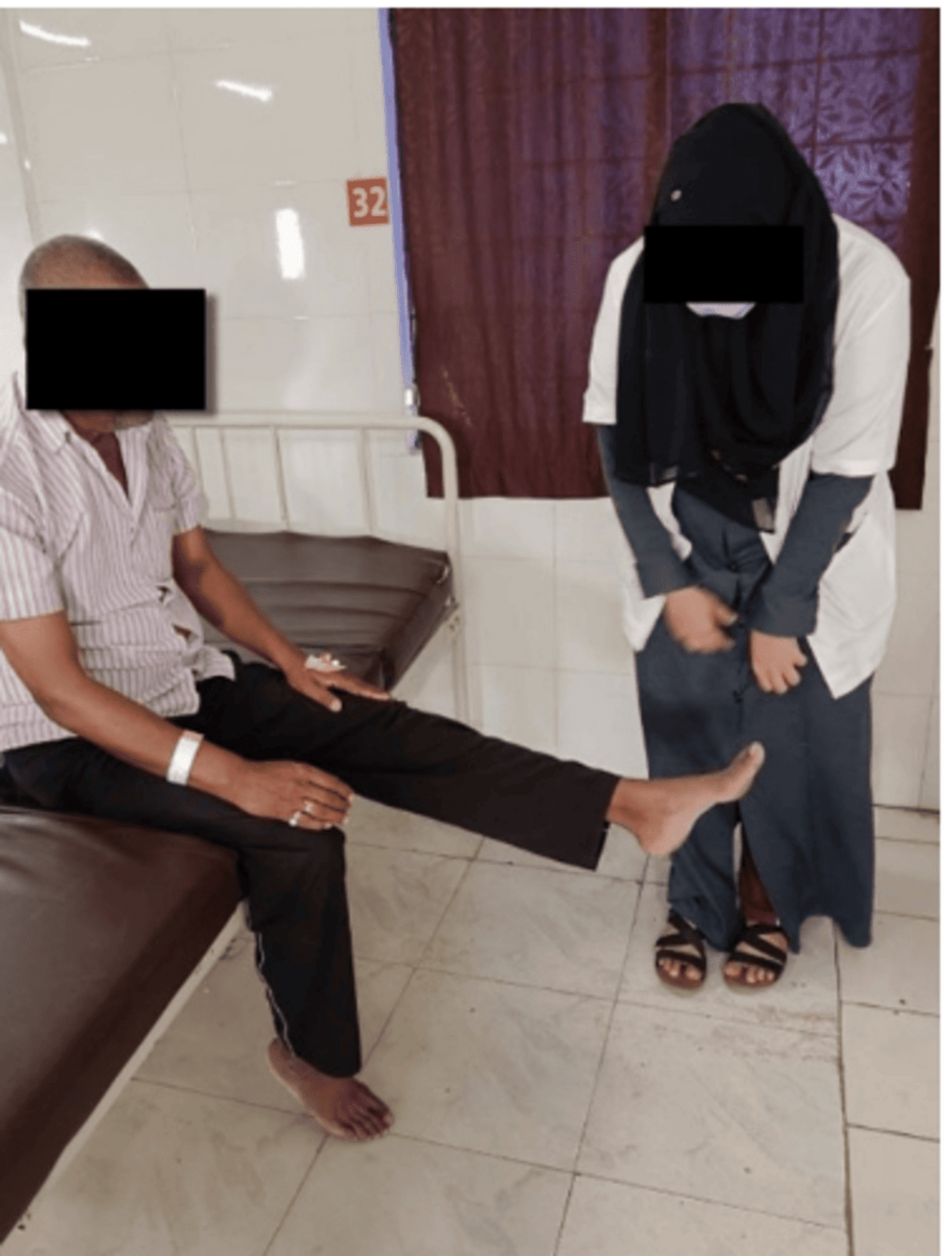
Patient performing dynamic quadriceps exercise

During dynamic quadriceps exercises, the patient actively and intentionally works at strengthening the quadriceps muscles of the anterior thigh. The goal of these exercises is a major component of physical therapy and rehabilitation programs that are devised to improve lower limb strength and function, as well as mobility in general. It is shown in Figure 4.

Outcome measure and follow-up

On assessment of muscle tone in the patient after 30 days of intervention, there were some notable improvements: the right upper limb had a tone of 1+, indicating slight improvement of muscle tone from the preintervention state, while the left upper limb had a rating for tone of 2+, showing a more significant improvement in the muscle tone. In the lower limbs, the right was increased to 1+, and the left was 2+. This may reveal the improvement in the upper and lower limbs' muscle tone after the intervention, with the left being more prominent. This outcome is summed up in Table 4.

**Table 4:** Table demonstrates variance in muscle tone

Muscle group	Post-intervention (30days) Right Limb	Post-intervention (30days) Left Limb
Upper Limb	1+	2+
Lower Limb	1+	2+

After 30 days of intervention, there was significant improvement in the strength of different muscle groups. The strength of right shoulder flexors, extensors, abductors, and adductors improved from 3/5 to 5/5, whereas elbow flexors and extensors, and wrist flexors also improved from 3/5 to 5/5. Wrist extensors improved from 2/5 to 4/5. Similarly, hip flexors and extensors improved from 2/5 to 4/5. Accordingly, knee flexors and extensors improved their strength from 3/5 to 5/5, while ankle dorsiflexors and plantar flexors improved their strength from 2/5 to 4/5. On the left side, shoulder flexors and extensors improved from 2/5 to 4/5, with the same improvement for shoulder abductors and adductors. The strength in elbow flexors and extensors, including wrist flexors, improved from 3/5 to 5/5. Wrist extensors improved from 3/5 to 5/5. The hip flexors and extensors increased significantly from 1/5 to 5/5. The knee flexors went from 2/5 to 4/5, and the knee extensors improved from 2/5 to 5/5. Both ankle dorsiflexors and plantar flexors improved from 2/5 to 4/5. In general, there was a significant increase in muscular strength on both sides after the treatment, although much more marked on the left. This is shown in Table 5.

**Table 5:** Table demonstrates the grades of manual muscle testing (MMT)

Muscle groups	Right Side Pre-intervention (day1)	Right Side Post-intervention (day 30)	Left Side Pre-intervention (day1)	Left Side Post-intervention (day 30)
Shoulder flexors	3/5	5/5	2/5	4/5
Shoulder extensors	3/5	5/5	2/5	4/5
Shoulder abductors	2/5	4/5	2/5	4/5
Shoulder adductors	2/5	4/5	2/5	4/5
Elbow flexors	3/5	5/5	3/5	5/5
Elbow extensors	3/5	5/5	3/5	5/5
Wrist flexors	3/5	5/5	3/5	5/5
Wrist extensors	2/5	4/5	3/5	5/5
Hip flexors	2/5	4/5	1/5	5/5
Hip extensors	2/5	4/5	1/5	5/5
Knee flexors	3/5	5/5	2/5	4/5
Knee extensors	3/5	5/5	2/5	5/5
Ankle dorsiflexors	2/5	4/5	2/5	4/5
Ankle plantar flexors	2/5	4/5	2/5	4/5

**Figure 5:**
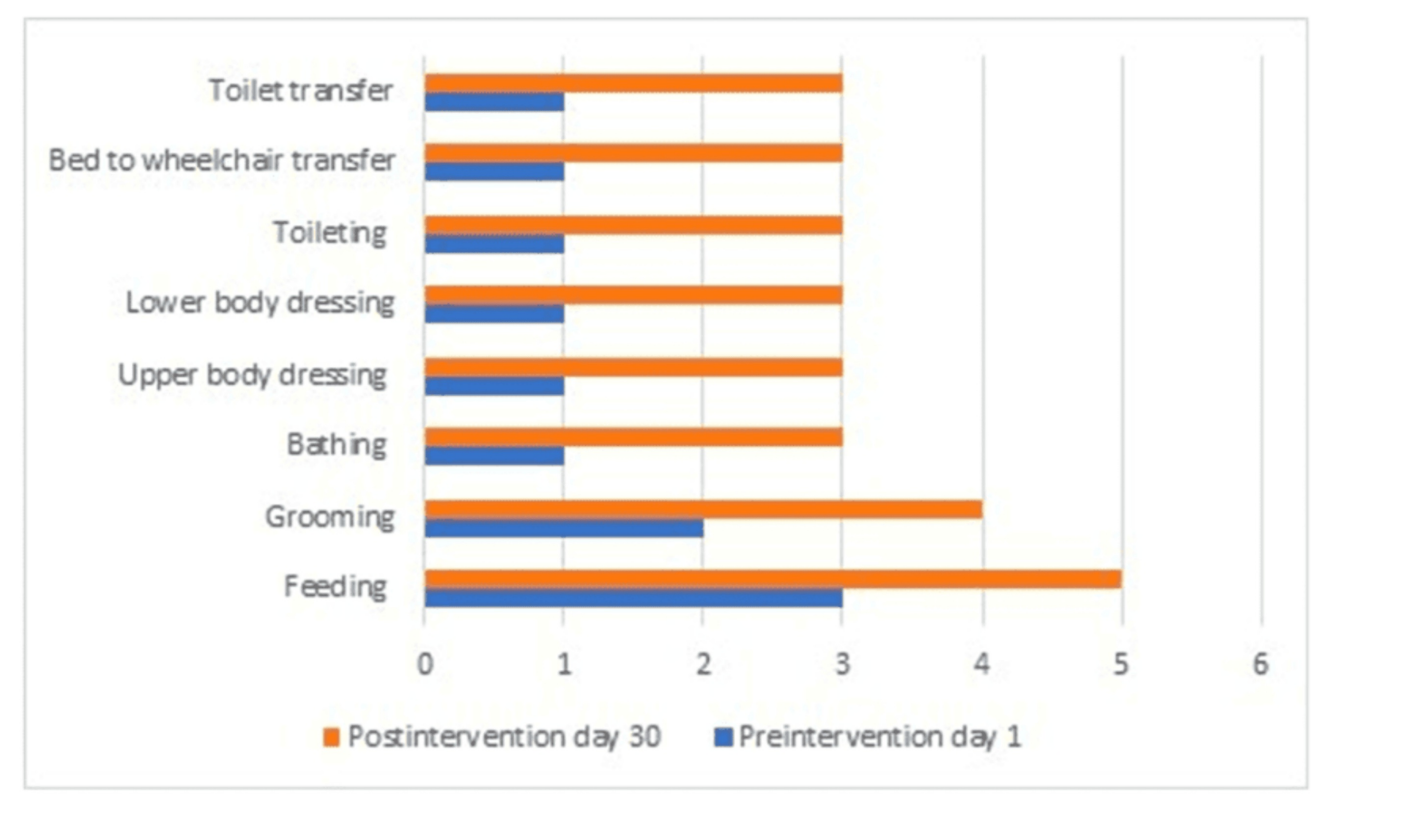
FIM scale: Orange colour represents postintervention, blue colour represents preintervention. FIM: Functional Independence Measure

The Functional Independence Measure (FIM) scale was used to measure the level of functionality of the patient before and after the intervention. Specifically, pre-intervention, the patient's scores indicated the following: Feeding component at level 3 and Grooming likewise at level 3. However, Bathing, Upper and Lower Body Dressing, Toileting, transfers from bed to wheelchair, and transfers to the toilet scored on the lowest level of 1, indicating complete dependence upon assistance to perform these activities. In the post-intervention, the patient showed a significant gain in functional independence. The Feeding component improved to level 5; this increase showed the patient's substantially improved state in feeding himself. Grooming improved to level 4, hence showing better skills in self-caring. Bathing, Upper and Lower Body Dressing, Toileting, and transfers from bed to wheelchair and also to the toilet were all improved to level 3; therefore, this reflected a moderate degree of independence with a lessened use of assistance. This is shown in Figure 5. 

## Discussion

Longitudinal extensive transverse myelitis (LETM) is defined by intramedullary spinal cord signal abnormalities that extend craniocaudally across at least three vertebral bodies and over two-thirds of the cross-sectional area on magnetic resonance imaging (MRI). Patients with LETM may present with significant clinical symptoms such as quadriparesis or paraparesis, gait abnormalities, and bladder, bowel, and sexual dysfunction [8,15]. The spinal cord lesion that spans three or more vertebrae on a spine MRI is known as LETM. A patient with LETM may appear clinically with significant symptoms such as quadriparesis or paraparesis, gait abnormalities, bladder, bowel, and sexual dysfunction [16,17]. According to Onyekere et al, in patients with acute transverse myelitis symptoms were alleviated by physical therapy [18].

In this case, managing patient rehabilitation is greatly aided by physical and rehabilitation medicine [19]. The rehabilitation session for the 62-year-old patient with longitudinal extensive transverse myelitis resulted in notable improvements in muscle tone and strength over a 30-day period. Post-intervention evaluations using the Functional Independence Measure (FIM) scale showed significant gains, with muscle tone improvements noted as 1+ in the right upper and lower limbs and 2+ in the left upper and lower limbs. Manual Muscle Testing (MMT) grades demonstrated substantial progress in muscle strength across various groups, demonstrating overall enhanced functional capability and muscle strength post-intervention. In a brief overview, physical rehabilitation is an essential component of the all-encompassing care of people with LETM. It provides a host of advantages, such as pain relief, increased mobility, increased independence, recovery driven by neuroplasticity, psychological counselling, and long-term management techniques [9]. For the purpose of optimizing functional recovery and enhancing quality of life, people with LETM must attend regular physical therapy sessions. Depending on the progress, goals, and continuing rehabilitation requirements of the individual, rehabilitation sessions may differ in frequency and length [7]. To guarantee a thorough and well-coordinated approach to rehabilitation, close coordination between the patient, their medical team, and the physical therapist is necessary [11].

## Conclusions

To sum up, the physical therapy intervention utilized in this case study proved to be quite successful. The patient's mobility was improved overall, and their strength, endurance as well as respiratory system improved. According to this case study, early and focused physical therapy intervention can greatly improve patients' quality of life and rate of recovery. These encouraging results highlight the important role that physical therapy plays in the management of longitudinal extensive transverse myelitis, underscoring its importance in the comprehensive care and rehabilitation of those who are affected.
